# Development of National Diagnostic Reference Levels for Chest Computed Tomography in Georgia

**DOI:** 10.7759/cureus.102125

**Published:** 2026-01-23

**Authors:** Irakli Tortladze, Otar Urushadze, Grigol Nemsadze, Nino Esvanjia, Ketevan Karanadze

**Affiliations:** 1 Radiology, Tbilisi State Medical University, First Medical University Clinic, Tbilisi, GEO; 2 Clinical Radiology, Tbilisi State Medical University, Tbilisi, GEO; 3 Medical Physics, LEPL Agency of Nuclear and Radiation Safety, Tbilisi, GEO; 4 Radiology, Aleksandre Aladashvili Clinic, Tbilisi, GEO

**Keywords:** alara, ct, drl, georgia national drls, radiology

## Abstract

Computed tomography (CT) is an essential diagnostic modality in modern medicine; however, it is associated with relatively high radiation exposure. Optimizing radiation dose while maintaining diagnostic image quality remains a major priority in clinical practice. Diagnostic reference levels (DRLs) are internationally recommended tools for dose optimization, yet national DRLs for chest CT examinations have not previously been established in Georgia.

The objective of this study is to assess radiation dose metrics from non-contrast chest CT examinations across medical institutions in Georgia and to establish preliminary national DRLs for adult and pediatric populations based on real-world clinical practice.

A nationwide, multicenter observational study was conducted across 27 Georgian medical institutions. Dose data from 136 consecutive patients who underwent non-contrast chest CT examinations between November 2024 and August 2025 were retrospectively analyzed. Both adult and pediatric patients were included. Radiation dose indicators, including CT Dose Index Volume (CTDIvol) and Dose Length Product (DLP), were collected alongside patient anthropometric parameters. Dose distributions were stratified by body mass index (BMI). DRLs were derived using the 75th percentile methodology in accordance with recommendations from the International Commission on Radiological Protection (ICRP).

Substantial inter-facility variability in CT radiation dose metrics was observed, reflecting differences in scanner technology, protocol design, and utilization of automatic exposure control (AEC) systems. BMI-stratified analysis enabled the derivation of realistic, country-specific reference values. The calculated 75th percentile values of CTDIvol and DLP served as the basis for defining preliminary national DRLs for non-contrast chest CT examinations in Georgia.

This nationwide assessment demonstrates the feasibility and importance of establishing national DRLs for chest CT in Georgia. The proposed DRLs provide a practical framework for radiation dose optimization without compromising diagnostic quality. Adoption of national DRLs is expected to reduce unnecessary radiation exposure, support continuous quality improvement, and enhance patient safety. Future efforts should focus on periodic DRL updates, broader implementation of dose-optimization technologies, and expansion to additional CT examination types.

## Introduction

Radiology and its advanced modality, computed tomography (CT), represent fundamental pillars of modern diagnostic medicine. CT has substantially enhanced the detection, characterization, and longitudinal evaluation of a wide range of pathologies; however, these diagnostic advantages are accompanied by radiation-related risks inherent to ionizing imaging. Exposure to high radiation doses may increase the probability of stochastic health effects, underscoring the importance of ensuring robust radiation protection for patients [1].

Contemporary radiation-protection standards are grounded in the three central principles established by the International Commission on Radiological Protection (ICRP): justification, optimization (as low as reasonably achievable (ALARA) - the guidance for radiation safety), and dose limitation. Optimization is particularly critical in CT imaging, given the modality’s comparatively higher dose burden. Although technological advancements enable meaningful dose reduction, effective implementation requires both properly functioning equipment and consistently managed examination protocols [2].

Among the technical factors influencing patient dose, automatic exposure control (AEC) plays a central role. AEC systems adjust tube current according to patient size, body habitus, and tissue attenuation, thereby reducing unnecessary exposure, ensuring appropriate mA modulation, and minimizing inter-operator and inter-device variability. Consequently, the correct configuration and regular use of AEC systems constitute essential components of radiation-dose optimization [3]. In Georgia, the standardization of radiological practice remains in an evolving phase. Healthcare facilities operate heterogeneous CT equipment - often of differing generations and calibration status - while protocol variability, uneven staff training, and partially aligned regulatory frameworks limit compliance with contemporary international standards. Addressing these challenges requires a system-level approach involving harmonization of technical infrastructure, regulatory mechanisms, workflow processes, and staff competencies [4].

Within this context, diagnostic reference levels (DRLs) play a pivotal role. Widely endorsed by organizations such as the International Atomic Energy Agency (IAEA) and ICRP, DRLs serve as practical tools for dose optimization, based on systematically collected local, regional, or national dose-distribution data. Their purpose is to identify unnecessarily high radiation doses and support continuous improvement in clinical practice.

The development of DRLs typically involves: defining dose indicators for specific examination types, determining DRL values and units, establishing standardized dose-measurement systems, aggregating large, representative datasets, and integrating DRLs into routine clinical workflow [5].

According to international guidance, DRLs are commonly derived from the 75th percentile of dose distributions collected from compliant patient cohorts. This statistical approach enables the identification of facilities operating at atypically high dose levels and encourages directed optimization efforts [6].

Importantly, DRLs do not constitute regulatory dose limits, nor do they demarcate “good” versus “poor” practice. Instead, they function as optimization benchmarks. Their application must not compromise diagnostic image quality, which remains the primary determinant of clinical appropriateness.

Georgia is currently in the process of establishing national DRLs - a complex but essential undertaking. Our research group is contributing to this effort through comprehensive data collection and analysis across a broad range of clinical facilities. As in other countries, Georgia requires its own DRLs because patient size, body mass index (BMI) distributions, equipment variations, and local clinical practices collectively influence achievable dose levels.

## Materials and methods

This multicenter, retrospective observational study was conducted across 27 medical facilities in Georgia. To ensure stable clinical workflows and representative radiation dose patterns, we included institutions that had provided CT services for a minimum of five years. The facility types included 20 multiprofile hospitals, three monoprofile medical centers, and four outpatient diagnostic facilities. The study population comprised 136 (n = 136) consecutive patients examined between November 2024 and August 2025. This sample included 87 (64%) males and 49 (36%) females, with ages ranging from one to 67 years.

Patients were included if they underwent non-contrast chest CT examinations performed for diagnostic purposes. Exclusion criteria were strictly applied to maintain data integrity: we excluded contrast-enhanced CT studies, examinations with incomplete dose documentation, repeated scans for the same clinical indication during a single visit, and studies affected by significant motion artifacts or protocol deviations.

Data were extracted from radiology department records and CT dose reports. We recorded patient weight, height, and BMI, alongside technical specifications. Primary radiation dose indicators were volume CT Dose Index (CTDIvol, measured in mGy) and DLP (measured in mGy · cm). Additional parameters included tube voltage (kV), and tube current-time product (mAs). Table 1 shows the radiological examination parameters across a subset of participating clinics.

**Table 1:** Radiological examination parameters across a subset of participating clinics. The table displays age, CTDIvol, DLP, and technical parameters (mAs, kV, BMI) for 16 representative cases.

Age	CTDIvol (mGy)	DLP (mGy · cm)	mAs	kV	BMI	mA	Clinic no.
1	3.7	74.1	231	120	12.6	50	#1
38	11	386.8	985	120	20.3	150	#2
69	11.4	476.4	1183	120	28.4	150	#3
92	15.2	460.50	1542	120	24.8	200	#4
58	14.6	479.30	1837	120	31.3	200	#5
72	11.9	466.21	-	120	24.6	300/256	#6
38	12.62	520.65	1358	130	25.4	206/54	#7
13	8.86	209.8	1335	130	-	149/112	#8
68	-	355.80	-	120	-	250/174	#9
74	7.84	244	2137	110	22.1	156	#10
78	7.84	235	2076	110	23.8	156	#11
32	14.50	591	3791	120	23.4	300	#12
34	4.30	108	-	120	25.1	150/96	#13
61	8.9	274.6	1542	120	23.6	157/95	#14
71	25.2	579.40	1666	120	24.7	330/148	#15
47	31.2	700.50	1930	120	22.5	388/239	#16

The sample size of 136 patients was determined as a representative pilot cohort to establish a baseline for national DRLs. While a formal power analysis for clinical efficacy was not the primary objective of this dose-audit study, the cohort size aligns with ICRP recommendations for initial DRL establishment in countries without prior national data. Descriptive statistics (mean, median, and 75th percentile) were calculated using standard statistical software. DRL values were specifically defined as the 75th percentile of the aggregated dose distributions, stratified by BMI to account for patient size variability.

## Results

**Figure 1:**
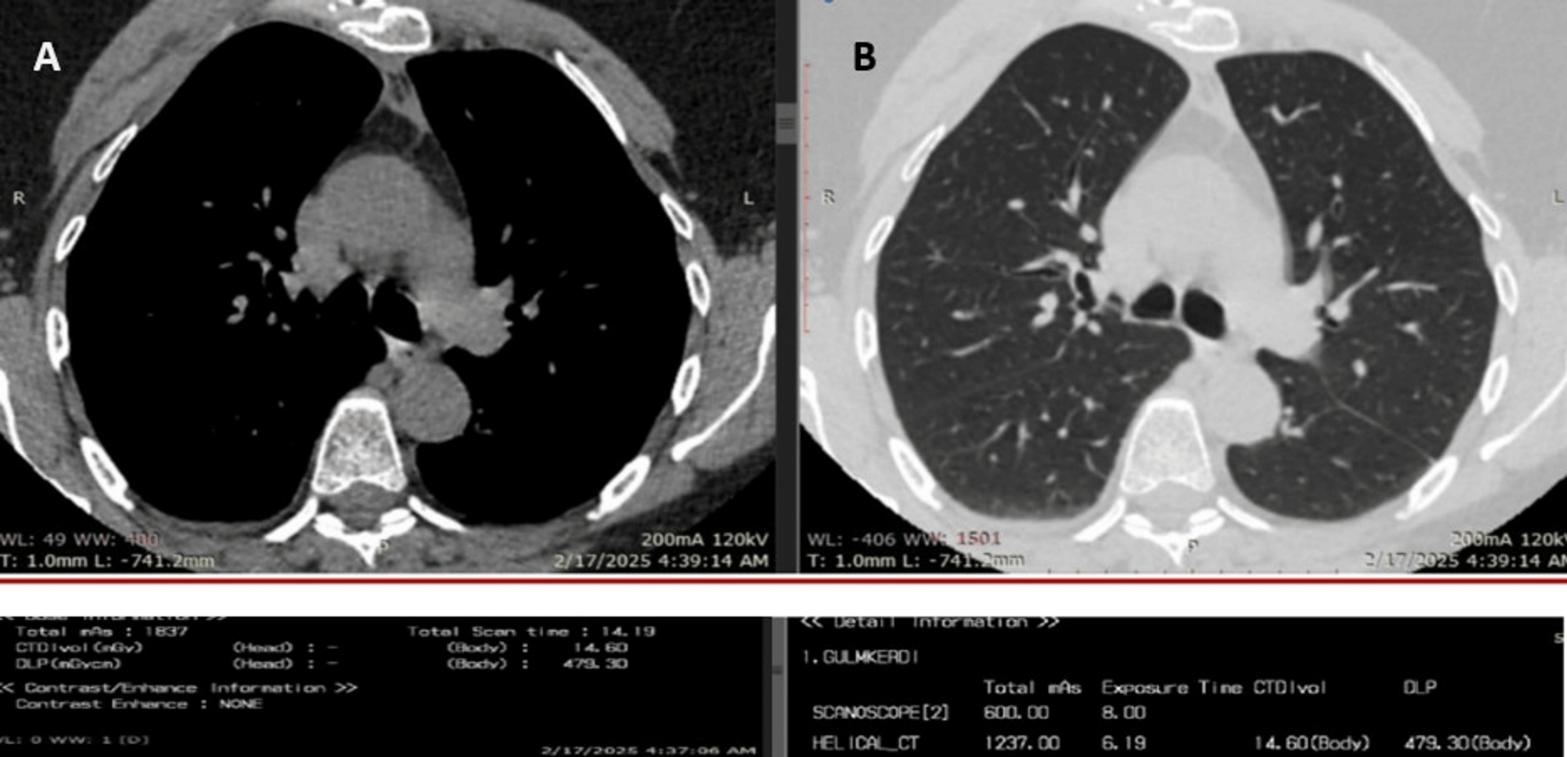
Adult chest CT scan illustrating dose metrics. (A) Soft tissue window and (B) lung window. The images demonstrate that diagnostic clarity is maintained at the recorded DLP

**Figure 2:**
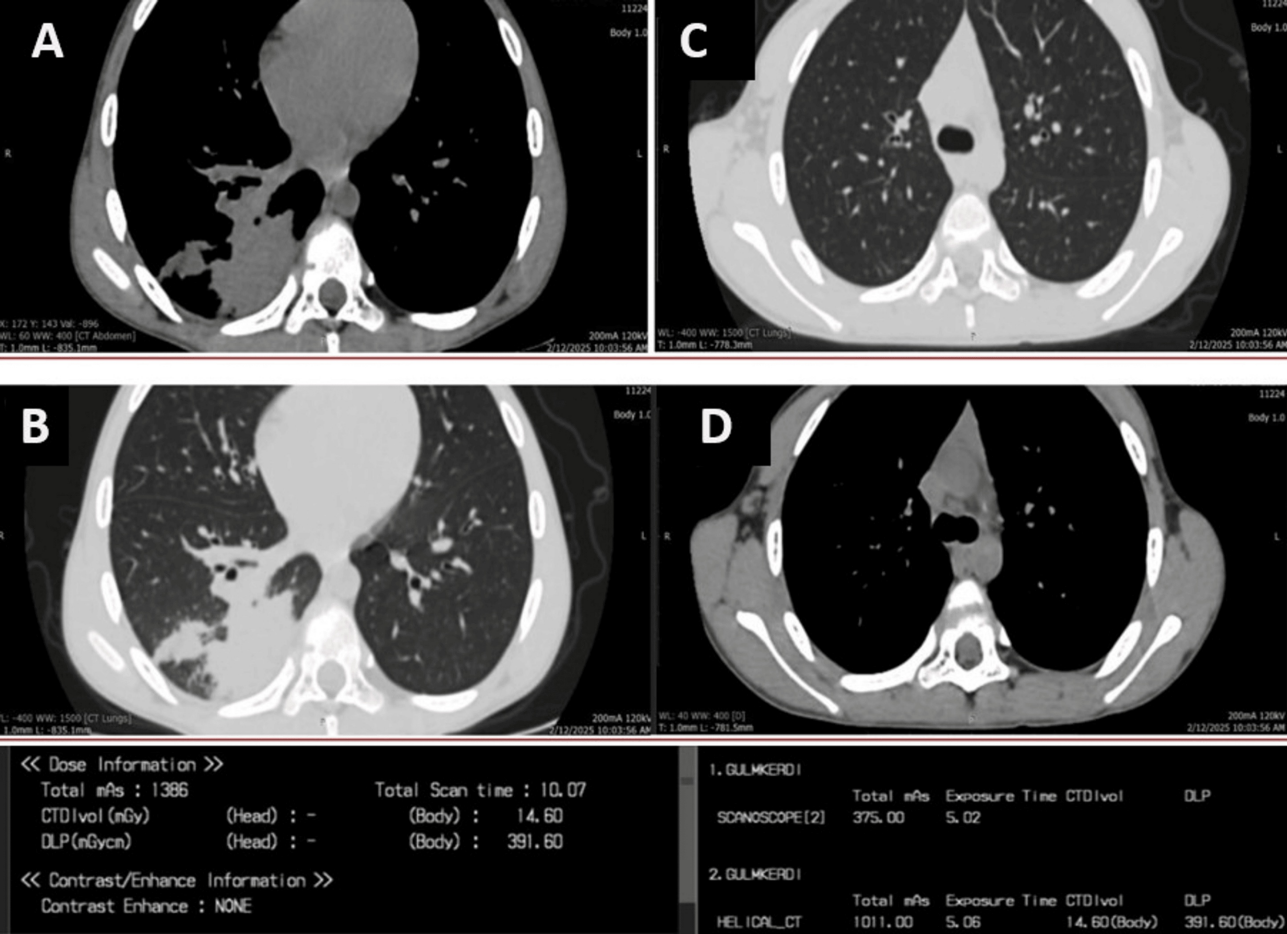
Pediatric chest CT scan showing clinical pathology. (A) Axial soft tissue window and (B) lung tissue window. Consolidative and infiltrative changes are visualized in the right lung despite lower dose parameters. (C) Axial lung window and (D) axial soft tissue window, no pathological changes.

**Figure 3:**
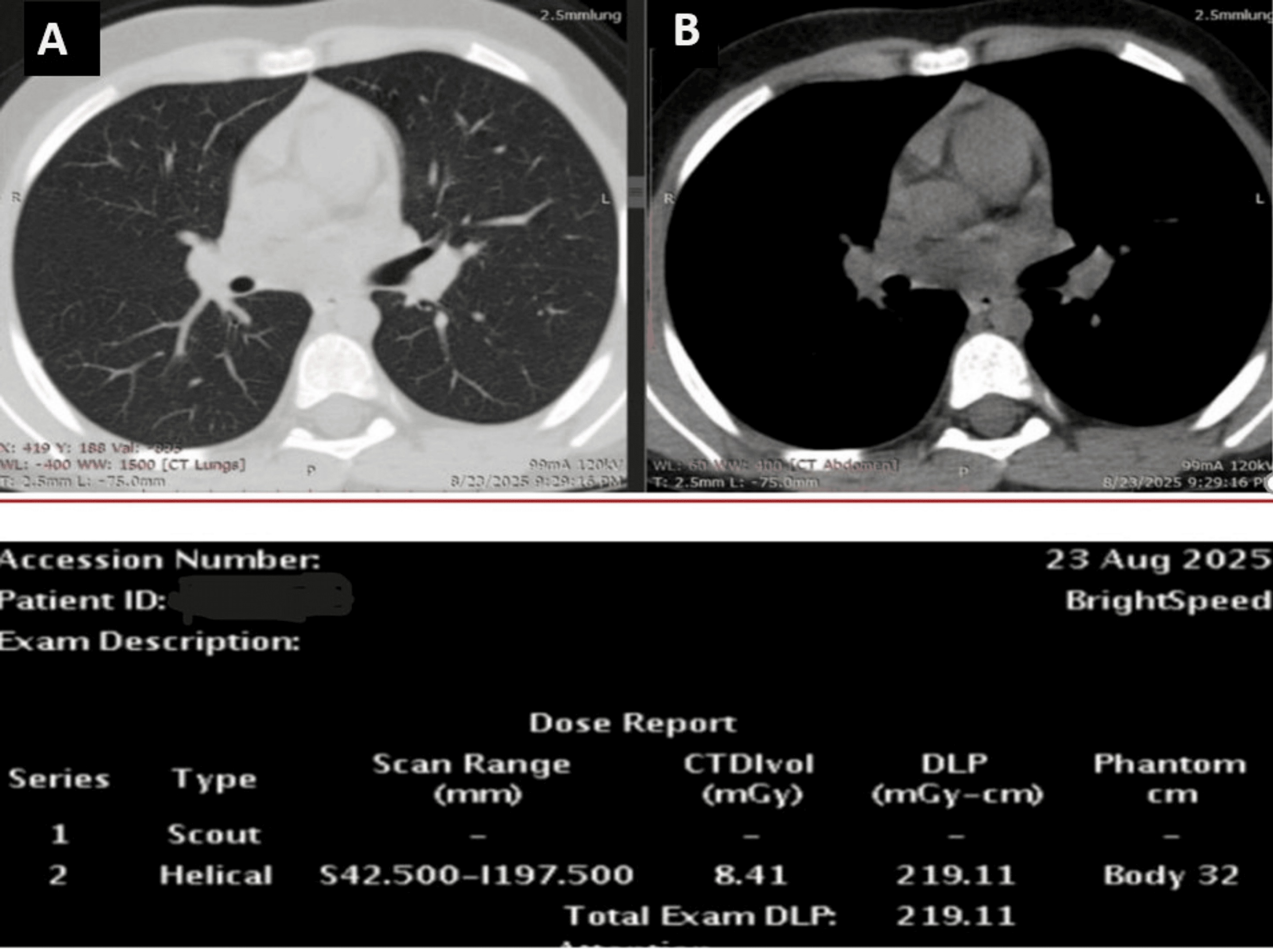
Another axial chest CT scan shows images in lung (A) and soft (B) tissue windows. A reduction in DLP is observed following the parameter adjustment

**Figure 4:**
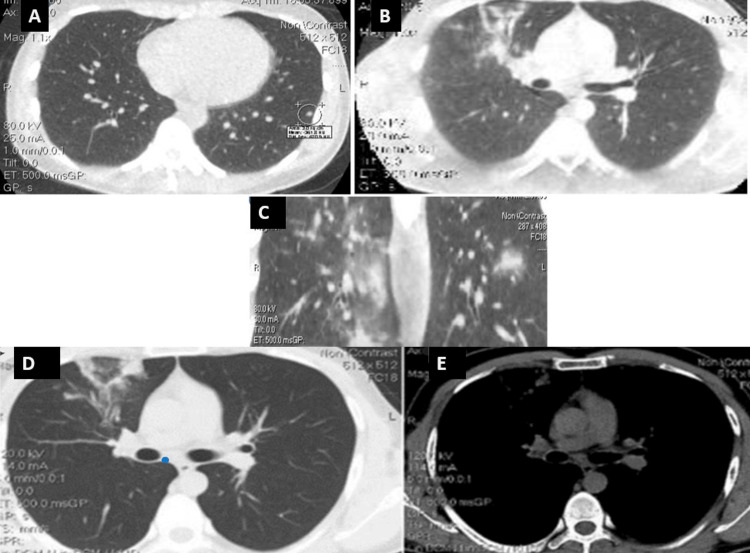
High-quality diagnostic imaging with modified low-dose protocols. (A,B,C,D) Lung window screening and (E) mediastinal window. These types of protocols are successfully used for repeat CT scans and hospitalization monitoring.

Representative chest CT images obtained from study patients are presented to illustrate the impact of protocol modifications on image quality and radiation dose (Figures 1-3). Images before and after parameter adjustments demonstrate that diagnostic assessment of the chest area remains feasible even when dose-optimization strategies are applied. Detailed evaluation of relevant anatomical structures was not compromised by the modified protocols (Figure 4).

All participating institutions exhibited variability in baseline CT dose metrics, reflecting differences in equipment generation, scan protocol design, and utilization of AEC systems. This inter-facility variability underscores the need for standardized national guidance and supports the rationale for establishing DRLs.

The study also highlights the importance of adopting ultra-low-dose CT examination practices where clinically appropriate. Implementing such protocols requires careful consideration of patient-specific parameters, including age, body habitus, and clinical indication. Our team utilizes these sensitive, diagnosis-specific protocols cautiously to ensure diagnostic adequacy while minimizing radiation exposure (https://www.gbmn.org/article120).

Descriptive statistics for non-contrast chest CT examinations showed a mean CTDIvol of 346.6 mGy, a median of 310 mGy, and a 75th percentile of 469 mGy. DLP and other technical parameters, including tube voltage (kV), tube current-time product (mAs), and estimated effective dose (E, mSv), were analyzed similarly and stratified according to BMI to inform DRL derivation.

To further clarify these findings, the observed CTDIvol and DLP distributions reflect real-world clinical practice across institutions with differing levels of protocol optimization. Higher dose values were predominantly associated with scanners lacking advanced dose-reduction features or with limited use of AEC. In contrast, facilities employing optimized protocols and size-adapted exposure parameters demonstrated lower dose metrics while preserving diagnostic image quality. The use of BMI (we would like to clarify that BMI classification in our study was based on the Medical Metabolic Specialists (MMS) classification) stratification allowed for more meaningful comparison across patient groups and supported the derivation of representative, population-adjusted DRL values. These results indicate that the identified dose variability is largely modifiable through protocol standardization and targeted optimization strategies rather than being inherent to diagnostic requirements.

## Discussion

This study revealed substantial inter-facility variability in CT radiation-dose metrics across Georgia, a finding that highlights the urgent need for national standardization. These differences primarily reflect variations in scanner technology, heterogeneous scan protocols, and inconsistent utilization of AEC systems. Similar sources of dose variability have been widely reported in multicenter CT dose audits and national DRL initiatives in other countries [7-10]. In many centers, AEC functionality is either underutilized or improperly configured, leading to unnecessarily high radiation exposure for patients with smaller body habitus, as previously demonstrated in international CT optimization studies [11,12].

When comparing our results with international data, the dose variability observed in Georgia mirrors patterns reported in regions that are in early phases of establishing national benchmarks, including parts of Eastern Europe and the Asia-Pacific region [13,14]. Studies from these areas consistently identify equipment heterogeneity and protocol variation as primary contributors to dose dispersion. Our proposed 75th percentile values (DRLs) therefore provide a practical and evidence-based starting point for Georgian facilities to audit their own performance and identify opportunities for optimization, in line with ICRP and IAEA recommendations [6,15].

The role of DRLs as optimization tools rather than regulatory limits has been emphasized in multiple international guidelines. DRLs are intended to identify unusually high dose levels and trigger protocol review, not to define acceptable or unacceptable clinical practice [5,16]. Importantly, adherence to DRLs must not compromise diagnostic image quality, which remains the primary determinant of clinical appropriateness. Previous studies have demonstrated that meaningful dose reductions in chest CT can be achieved without loss of diagnostic confidence when protocol optimization is guided by DRLs and patient-specific parameters [17-19].

Limitations

Several limitations must be acknowledged. First, the sample size of 136 patients is relatively modest for a nationwide study, which may limit the generalizability of the findings to very rare scanner types. Second, the study focused exclusively on non-contrast chest CT; therefore, these DRLs cannot be applied to contrast-enhanced or abdominal studies. Finally, while BMI stratification was performed, more granular patient factors like specific clinical indications (e.g., screening vs. acute trauma) were not fully integrated into the statistical model.

Future directions

Future efforts should focus on expanding data collection to include other common CT examinations, such as head and abdomen scans. We recommend the full implementation of AEC systems across all institutions and the integration of DRLs into electronic dose-tracking platforms. Regular updates of these reference levels every three to five years will ensure they reflect the latest technological advancements in radiology [10,13].

## Conclusions

This nationwide assessment of non-contrast chest CT radiation doses represents the first step toward establishing national DRLs in Georgia. The study demonstrates substantial inter-facility variability in CT dose metrics, influenced by differences in equipment, protocol design, and utilization of AEC systems. BMI-stratified analysis and the application of the 75th percentile methodology enabled the derivation of realistic, country-specific reference values aligned with international recommendations.

The proposed DRLs provide a practical framework for radiation dose optimization while preserving diagnostic image quality. Their adoption is expected to enhance patient safety, promote quality improvement, and support harmonization of radiological practice nationwide. Future efforts should focus on expanding DRLs to other CT examinations, strengthening quality assurance programs, and regularly updating reference levels in response to technological advancements.
